# New insights of metabolite abnormalities in the thalamus of rats with iminodiproprionitrile-induced tic disorders

**DOI:** 10.3389/fnins.2023.1201294

**Published:** 2023-09-29

**Authors:** Jingru Yu, Xuan Yao, Xin Zhang, Juanjuan Hao

**Affiliations:** ^1^School of Medicine, Shaoxing University, Shaoxing, China; ^2^Xin Hua Hospital Affiliated to Shanghai Jiao Tong University School of Medicine, Shanghai, China

**Keywords:** tic disorders, glutamate, γ-aminobutyric, metabolic loop, widely targeted metabolomic analysis, neurosteroids

## Abstract

**Introduction:**

This study aimed to investigate pathological changes in the “Glutamate (Glu)-γ-aminobutyric acid (GABA)” loop and apply widely targeted metabolomic analysis technology to comprehensively explore metabolite abnormalities/ in the thalamus of rats with tic disorders (TD).

**Methods:**

Wistar rats were randomized into control, TD, and tiapride (Tia) groups. Iminodipropionitrile (IDPN) was used to induce TD in rats. The Tia group was administered tiapride. Neurotransmitter levels in the thalamus of rats in the three groups were measured using UPLC-3Q MS. And, the protein expression levels of Glu decarboxylase (GAD65/67) and GABA transporter protein (GAD-T) were measured using western blotting. The mRNA expression levels of these genes were evaluated using real-time polymerase chain reaction. Lastly, other metabolites in the thalamus were detected by widely targeted metabolomic analysis between TD and Control group rats.

**Results:**

The Glu level, Glu/GABA ratio, and Asp level in the TD group were significantly higher (all *p* < 0.001) than those of the Control group, whereas the GABA and Gly levels were lower (*p* < 0.001 and *p* = 0.009, respectively). The Tia group exhibited a significant reduction in the Glu level (*p* = 0.001) compared with the TD group. The protein expression level of GAD67 in TD group was higher (*p* = 0.009) and the mRNA expression levels of GAD65, GAD67, and GAT-1 were lower (*p* < 0.05) than those of the Control group. The Tia group did not display any differences in GAD65, GAD67, or GAT-1 expression. Widely targeted metabolomic analysis revealed that 34 substances were abnornal between the TD and Control groups (9 upregulated and 25 downregulated). Neurosteroids (progesterone, corticosterone) exhibited distinct differences. Metabolite analysis using the Kyoto encyclopedia for genes and genomes indicated that the steroid hormone biosynthesis pathway may be involved in TD pathogenesis.

**Conclusion:**

This study revealed metabolic abnormalities in the thalamus of rats with TD. The interaction between neurotransmitters and neurosteroid biosynthesis represents a new direction for future studies.

## 1. Introduction

Tic disorders (TD) is a neuro-developmental disorder which is characterized by sudden, rapid recurrent, nonrhythmic movements (motor tics) and/or vocalizations (vocal tics; Leckman, 2012; Yang et al., 2022). Tics generally occur in childhood, with the severity peaking around the age of 10 and 12 years, and then recede during puberty, but some persist into adulthood (Xu et al., 2020; Ricketts et al., 2022). The large majority of patients with TD also present with one or more co-morbid psychiatric disorders, such as obsessive-compulsive disorder (OCD), attention deficit/hyperactivity disorder (ADHD), autism spectrum disorder (ASD), rage attacks, learning disorders and executive dysfunction (Szejko et al., 2022). TD, especially with severe tics and psychiatric comorbidities, have considerable negative affect to the quality of life, social functioning and academic achievement of patients (McGuire et al., 2013; Eapen et al., 2016; Pérez-Vigil et al., 2018; Johnson et al., 2023). And one investigation reported TD is associated with an increased risks for obesity, type 2 diabetes, and circulatory system diseases from childhood (Brander et al., 2019), and also a increased risk of premature death (Meier et al., 2017). However, TD has not received enough attention and it urgently needs to be studied in depth.

The pathophysiological mechanism of TD is complicated and has not been fully elucidated. Dysfunctions of the “cortico-striatal-thalamic-cortical” network have been suggested as the key pathogenesis of TD (Naro et al., 2020). Most of the data from animal experiments support the hypothesis of dopamine (DA) and 5-hydroxytryptamine (5-HT) system dysfunction (Wong et al., 2008; Maia and Conceição, 2018). Previous studies also reported the abnormal changes of amino neurotransmitter levels in the rodent brain (Zhang et al., 2020). However, to date, little attention has been paid to the metabolic circuits involving interconversion between neurotransmitters. Glutamate (Glu) is the main excitatory amino neurotransmitter, whereas γ-aminobutyric acid (γ-GABA) is inhibitory (Reiner and Levitz, 2018; Zhu et al., 2018). The “Glu-GABA” metabolic loop plays an important role in maintaining homeostasis and balance of neurotransmitters. Glu can be converted to GABA by glutamic acid decarboxylase (GAD; Hagan et al., 2022). GAD possesses two isoenzymes, GAD65 and GAD67, encoded by GAD1 and GAD2, respectively, (Jiang et al., 2022). The main transporter of GABA is aminobutyric acid transporter (GAT-1) encoded by SCL6A1 (Motiwala et al., 2022). GAT-1 is mainly responsible for the uptake and inactivation of GABA. In addition to these neurotransmitters, other metabolites in the brain may be involved in TD. These metabolites include nucleotides and their metabolites, organic acids and their derivatives, amino acid derivatives, hormones, hormone-related compounds, and phosphoric acids, and so on. Recently, metabolomic methods have been widely used to comprehensively analyze metabolites. Widely targeted metabolomics integrates the advantages of non-targeted and targeted metabolite detection technologies, achieves high throughput, and exhibits high sensitivity, precision, and wide coverage (Chen et al., 2020).

3,3-Iminodipropionitrile (IDPN), a synthetic nitrile, could induce abnormal movements in rodents, including spontaneous head movement, abnormal circling, backward movement, and sensory disturbances (Papasozomenos et al., 1981; Cadet, 1989; Tariq et al., 1999). As these abnormal movements in rodents resemble symptoms of TD in humans (Cadet et al., 1987a,b), animal model of IDPN is of particular importance to investigate the biochemical mechanisms of TD, and to develop future clinical therapeutics (Tóth et al., 1998). IDPN is commonly used to induce TD, which has a long-term effect (Chen et al., 2019; Lin et al., 2021; Wang et al., 2022, 2023).

Therefore, this study firstly observed the pathological changes in the “Glu-GABA” metabolic loop in the thalamus of rats with TD induced by IDPN. We additionally explored the potential therapeutic mechanism of tiapride which is a selective dopamine D2/D3-receptor antagonist (Peters and Faulds, 1994) and used to treat TD (Roessner et al., 2022). Lastly, we applied Widely targeted metabolomics technology to comprehensively detect and analyze metabolite abnormalities in TD.

## 2. Materials and methods

### 2.1. Animals

Male Wistar rats (Hangzhou, China), weighing approximately 50 g and 4 weeks of age at the beginning of the experiments, were used after 7-day adaptive breeding. The animals were housed under standard conditions with water and food *ad libitum*. All animal procedures were approved by the Ethics Committee on Animal Experiments of Shanghai Jiao Tong University School of Medicine (XHEC-F-2023-005). Animal pain was minimized during the experiment.

The rats were randomly divided into a Control group (*n* = 8) and a TD group (*n* = 16) after 1 week. The Control group was injected with 0.9% saline (15 mL/kg), while the TD group was injected with 3,3’-Iminodipropionitrile (IDPN, 250 mg/kg; Sigma-Aldrich Co, St Louis, MO, United States). After 7 days, we assessed the behavior of TD by evaluating the Stereotyped behavior scores.

The TD group was further divided into two groups: TD (*n* = 8) and Tia (*n* = 8). The Tia group was administered tiapride (21 mg/kg; Jiangsu Nhwa Pharmaceutical Co., Xuzhou, People’s Republic of China) by gavage once daily for 3 weeks. The control and TD groups were administered 0.9% saline (10 mL/kg).

### 2.2. Behavioral recording

#### 2.2.1. Open field test

The open field test was used to comprehensively evaluate loco motor and fear-related behaviors. The rats were placed in an open field test box (100 × 100 × 50 cm); the peripheral wall was black, and the bottom surface was divided into 25 square open fields of equal area. Behavior was recorded for 5 min using a camera. The open field test scores were the sum of the horizontal activity scores and vertical exercise scores. Frequences of horizontal and vertical behaviors were scored. Horizontal behaviors were considered when the rats climbed a grid (all four paws entered the grid), and each grid climbed was counted as 1 score. Vertical behaviors were considered when the rats raised their hind limbs, either by upright (both vacated or climbed the wall), and each upright was counted as 1 score.

#### 2.2.2. Stereotypy recording

Stereotyped behavior was classified as follows: mouth and paws movement, self-biting, spinning, head shaking (many swings from left to right and up and down), dance-like movement and licking and biting the cage. Stereotyped behavior score was rated as follows. No stereotyped behavior, 0; rotational behavior, (1) excessive up and down movements of the head and neck, (2) excessive up and down movements of the head and neck plus rotational behavior, (3) lateral head swing and excessive up and down movements of the head and neck, (4) The rats were placed in an open-field test box in a quiet, dark environment. Observations were made every 5 min for 1-min periods, for a total of 12 periods.

### 2.3. UPLC-3Q MS detection for amino acid neurotransmitters in the thalamus

The procedure was modified from a previous report (Hao et al., 2020). Briefly, rats were put into a small animal anesthesia machine (RWD Life Science Co., Ltd., Shenzhen, China) and euthanized with isoflurane. Then the thalamus in the brain tissue was immediately removed and placed in a weighed EP tube. The thalamus sample was completely homogenized by adding chilled magnetic bead lysate (375 μL) to the EP tube and allowing to stand for 120 s. The thalamus samples were centrifuged at 12,000 ×*g* for 15 min at 4°C. The supernatant was placed in a new EP tube. Then, 150 μL lysate was added to the precipitate, shaken, and centrifuged at 12,000 g for 5 min at 4°C. The supernatant was then removed and mixed with the previous supernatant. Subsequently, chloroform (300 μL) was added to the precipitate, shaken, and allowed to stand for 30 min, at −20°C before being centrifuged at 12,000 g for 20 min at 4°C. The supernatant was removed, mixed with the supernatant mixture, and diluted five-fold to obtain the solution for analysis.

UPLC-3Q MS neurochemistry analyses were conducted using ultra-performance liquid chromatography (H-Class UPLC) coupled with Xevo TQ-XS triple quadrupole MS (Waters, Milford, MA, United States). Separation was obtained with an Acquity UPLC BEH Hilic column (2.1*100 mm i.d., 1.7-μm particle size; Waters, Milford, MA, United States) at ambient temperature using 0.1/100 mL ammonium formate in water (eluent A) and 0.1/100 mL formic acid in acetonitrile (eluent B) as the mobile phase at a constant flow rate of 0.4 mL min^−1^. The compounds were eluted using the following gradient profile: 0 min,10% A; 1 min, 20% A; 5 min, 50% A; 10 min, 90% A; 12 min, 10% A; and 14 min, 10% A. The injection volume was 1 L. The electron spray ionization (ESI) source used in this study was operated in positive ion mode, and its main working parameters were as follows: capillary voltage: 3.0 kV; source temp: 150°C; desolvation temp: 400°C; and desolvation gas: 800 L/h. Ion pairs were detected in the multiple reaction monitoring (MRM) mode. The m/z transition pairs (precursor ion/product ion) were 515.0/497.1 TLM, 357.0/134.1, and 358.3/135.2. The quadrupoles were set at unit resolution. The analytical data were processed using the Masslynx software. Neurotransmitters were quantified relative to internal standard areas and calibrated using standard curves.

### 2.4. Western blot for GAD65, GAD67, and GAT-1

The thalamus (*n* = 6 per group) was homogenized and lysed using RIPA (50 mmol/L Tris•HCl pH8.0, 150 mmol/L NaCl, 1%SDS, 1 mmol/L PMSF). The protein concentration was quantified using the Bradford method. The protein was used for Western blotting with GAD65 (1:5,000, ab239372, Abcam, Cambridge, United Kingdom), GAD67 (1:1,000, ab213508, Abcam, Cambridge, UK), and GAT-1 (1:1,000, ab177483, Abcam, Cambridge, United Kingdom) primary antibody, incubated overnight at 4°C. After incubation, the membranes were washed thrice with Tris-buffered saline containing Tween 20 and then incubated with the secondary antibody (1:10,000) at room temperature for 1 h, and washed with TBST six times for 3 min each. The blots were visualized using enhanced chemiluminescence (WBKLS0500, Millipore). The electrochemiluminescence signals were detected using Total Lab Quant V11.5 (Newcastle upon Tyne, United Kingdom). β-Actin (A20120A0702, BioTNT) was used as an internal control to validate the amount of protein loaded onto the gels.

### 2.5. Real-time PCR for GAD65, GAD67, and GAT-1

The samples of thalamus tissue (*n* = 6 per group) were frozen at −80°C. The total RNA of the thalamic tissue samples was isolated using TRIzol reagent according to the manufacturer’s protocol (CWbio. Co. Ltd., Cat#CW0581). The primers used were as follows: GAD67 forward (TGCTGTTGCTGCGTTTTAGAG) and reverse (CCCCCTGCCCAAAGATAGA); GAD65 forward (CAGAATGATGGAGTATGGGAC) and reverse (GCGGAAGAAGTTGACCTTAT); and GAT-1 forward (GACCCTCCACTGTGTCTCGT) and reverse (AACCTCCTGCCTTCATCCTAA). For real-time PCR, total RNA was initially denatured at 95°C for 10 min, and then amplified for 40 cycles. For cDNA amplification, initial denaturation at 95°C for 5 min, followed by 40 cycles of denaturation at 95°C for 5 s, annealing and extension at 60°C for 30 s. The ABI7500 real-time PCR machine (Applied Biosystems, Foster City, CA, United States) was used to detect SYBR green signals. Gel electrophoresis and melting curve analysis were used to analyze the PCR products and confirm specific amplifications. The 2^−ΔΔCT^ value method was used to quantify the transcript levels.

### 2.6. Widely targeted metabolomic analysis for other metabolites

#### 2.6.1. Chemicals and reagents

HPLC-grade acetonitrile (ACN) and methanol (MeOH) were purchased from Merck (Darmstadt, Germany). Milli-Q water (Millipore, Bradford, United States) was used for all experiments. All standards were purchased from Sigma-Aldrich (St. Louis, MO, United States) and Zhenzhun. Formic acid was purchased from Sigma-Aldrich (St. Louis, MO, United States). Stock solutions (1 mg/mL) were prepared in MeOH and other solutions from the standards. All stock solutions were stored at −20°C. The stock solutions were diluted with MeOH to obtain working solutions before analysis.

#### 2.6.2. Sample preparation and extraction

After the sample was thawed and crushed, 0.05 g was mixed with 500 μL of 70% methanol/water. The sample was vortexed for 3 min at 2,500 r/min and centrifuged at 12,000 r/min for 10 min at 4°C. Then, 300 μL of supernatant was placed in a new centrifuge tube and stored in a refrigerator at −20°C for 30 min. The supernatant was recentrifuged at 12,000 r/min for 10 min at 4°C. After centrifugation, 200 μL of supernatant was transferred to a protein precipitation plate for further analysis using liquid chromatography coupled with mass spectrometry.

#### 2.6.3. UPLC conditions

The sample extracts were analyzed using an LC-ESI-MS/MS system (UPLC, ExionLC^1^; MS, QTRAP® 6,500+ System).^2^ The analytical conditions were as follows: T3 method˖HPLC: column (Waters ACQUITY UPLC HSS T3 C18; 100 mm × 2.1 mm i.d.ˈ1.8 μm); solvent system, water with 0.05% formic acid (A); ACN with 0.05% formic acid (B). The gradient was initiated at 5% B (0 min), increased to 95% B (8–9.5 min), and finally ramped back to 5% B (9.6–12 min); flow rate, 0.35 mL/min; temperature, 40°C; injection volume: 2 μL.

Amide method: HPLC column, ACQUITY UPLC BEH Amide (i.d.2.1 × 100 mm, 1.7 μm); solvent system, water with 10 mM ammonium acetate and 0.3% ammonium hydroxide (A), 90% ACN/water (V/V) (B); The gradient was initiated at 95% B (0–1.2 min), decreased to 70% B (8 min), 50% B (9–11 min), and finally ramped back to 95% B (11.1–15 min); flow rate, 0.4 mL/min; temperature, 40°C; injection volume: 2 μL.

#### 2.6.4. ESI-MS/MS conditions

Linear ion trap and triple quadrupole (QQQ) scans were acquired using a triple quadrupole-linear ion trap mass spectrometer (QTRAP), QTRAP® 6,500+ LC–MS/MS System, equipped with an ESI Turbo IonSpray interface, operating in both positive and negative ion modes, and controlled by Analyst 1.6.3 software (Sciex). The ESI source operation parameters were as follows: ion source, ESI+/−; source temperature 550°C; ion spray voltage, 5,500 V (positive), and-4500 V (negative); curtain gas was set at 35 psi. Metabolites were analyzed using scheduled MRM. Data acquisition was performed using Analyst 1.6.3 software (Sciex). Multiquant 3.0.3 software (Sciex) was used to quantify all metabolites. Mass spectrometry parameters, including the declustering potentials (DP) and collision energies (CE) for individual MRM transitions, were done with further DP and CE optimization. A specific set of MRM transitions was monitored for each period according to the metabolites eluted within the corresponding period.

#### 2.6.5. Detection of targeted metabolites

All those metobolites were detected by MetWare^3^ based on the AB Sciex QTRAP 6500 LC–MS/MS platform.

### 2.7. Statistical analysis

All data were expressed as mean ± SEM. The software package SPSS 25.0 (SPSS, United States) was used for data analysis. Signifcance of diferences among groups was determined using the analysis of variance (ANOVA) with the least signifcant diference (LSD). Signifcance was set at *p* < 0.05.

## 3. Results

### 3.1. Comparison of open field test and stereotyped scores of rats

**Figure 1:**
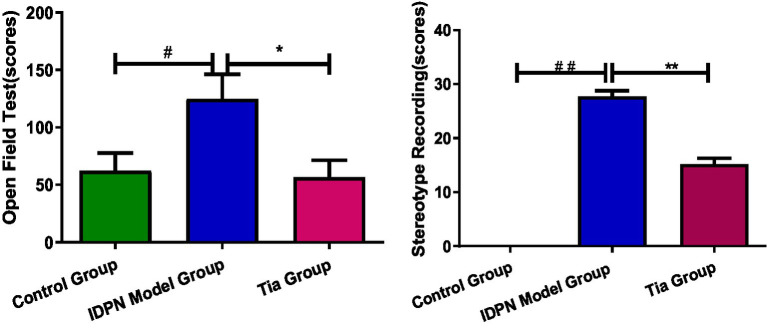
Behavioral recording Comparison of three groups. Compared with the Control group, ^##^ means *p* < 0.01, ^#^ means *p* < 0.05; compared with the TD group induced by IDPN, * means *p* < 0.05, ** means *p* < 0.01.

The open-field test scores of the TD group were significantly higher than those of the Control group (*p* < 0.05). After the intervention, the open-field test scores of the Tia group were significantly lower than those of the TD group (*p* < 0.05; Table 1; Figure 1).

**Table 1:** Comparison of the behavioral recording in three groups.

Group	The open-field test scores	The stereotype scores
Control group	62.2 ± 48.8	0
TD group induced by IDPN	124.9 ± 67.3^**#**^	27.70 ± 3.40^**##**^
Tia group	56.6 ± 47.0*	15.2 ± 3.29**

Compared with the Control group, ^##^means *p* < 0.01, ^#^means *p* < 0.05; compared with the TD group induced by IDPN, *means *p* < 0.05, **means *p* < 0.01.

The stereotype scores of the TD group were significantly higher than those of the Control group (*p* < 0.01). After the intervention, the stereotype scores of the Tia group were significantly lower than those of the TD group (*p* < 0.01; Table 1; Figure 1).

### 3.2. Comparison of the main amino acid neurotransmitters in the thalamus

**Figure 2:**
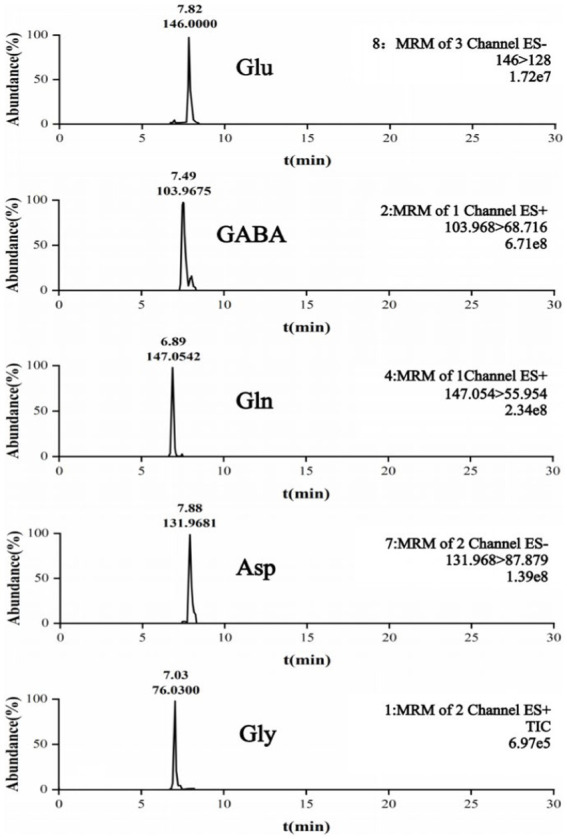
UPLC-3QMS chromatograms.

We used UPLC-3Q MS to detect amino acid neurotransmitters. We used an Acquity UPLC BEH Hilic column to separate the five analytes. The results are shown in Figure 2.

**Figure 3:**
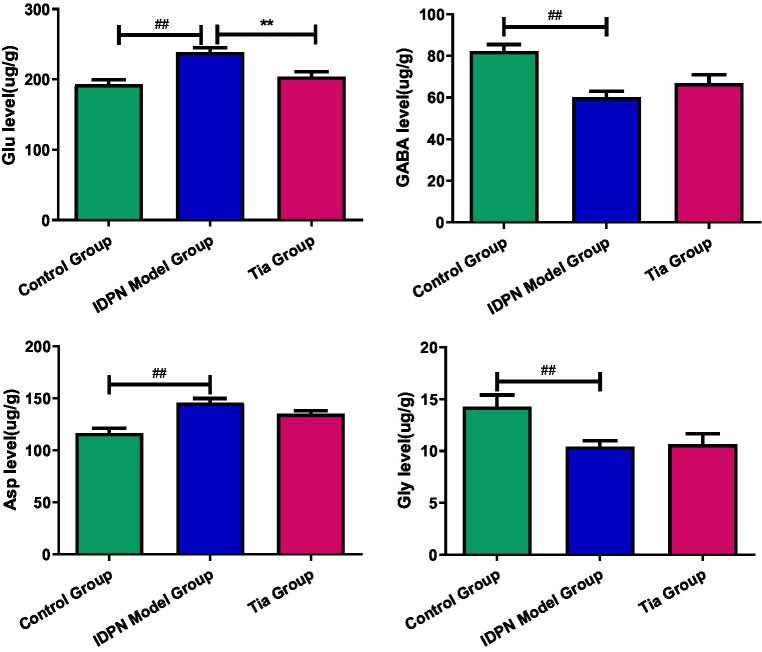
Comparison of the Glu, GABA and Asp level in the three groups. Compared with the Control group, ^##^ means *p* < 0.01; compared with the TD group induced by IDPN, ** means *p* < 0.01.

Compared with the Control group, in the TD group, the Glu level, Glu/GABA ratio, and Asp level were significantly increased (all *p* < 0.001), whereas the GABA and Gly levels were significantly decreased (*p* < 0.001 and *p* = 0.009, respectively). There were no statistically significant differences in the Gln levels among the three groups. The Glu level in the Tia group was significantly decreased compared to that of the TD group (*p* = 0.001). The results are presented in Table 2 and Figure 3.

**Table 2:** Comparison of the main amino acid neurotransmitters in the three groups.

Group	Glu (ug/g)	GABA (ug/g)	Gln (ug/g)	Glu/GABA	Asp (ug/g)	Gly (ug/g)
Control group	192.78 ± 19.50	82.31 ± 8.98	1.83 ± 0.13	2.38 ± 0.44	116.54 ± 12.63	14.27 ± 3.25
TD group induced by IDPN	238.77 ± 17.17^**##**^	60.08 ± 8.39^##^	1.68 ± 0.24	4.04 ± 0.64^##^	145.75 ± 11.47^##^	10.41 ± 1.72^##^
Tia group	204.19 ± 18.76^**^	66.75 ± 11.66	1.45 ± 0.28	3.18 ± 0.0.83^##^	134.92 ± 8.44	10.65 ± 2.86

Compared with the Control group, ^##^means *p* < 0.01; compared with the TD group induced by IDPN, **means *p* < 0.01.

### 3.3. Comparison of the protein expression level of GAD65, GAD67, and GAT-1 in “Glu-GABA” metabolic loop

**Figure 4:**
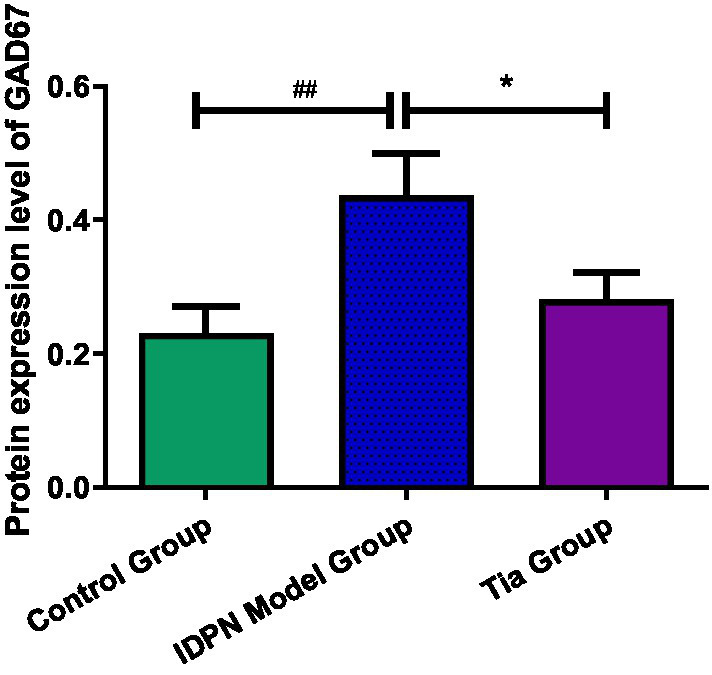
Comparison of GAD67protein expression in the three groups. Compared with the Control group, ^##^ means *p* < 0.01; compared with the TD group induced by IDPN, * means *p* < 0.05.

**Figure 5:**
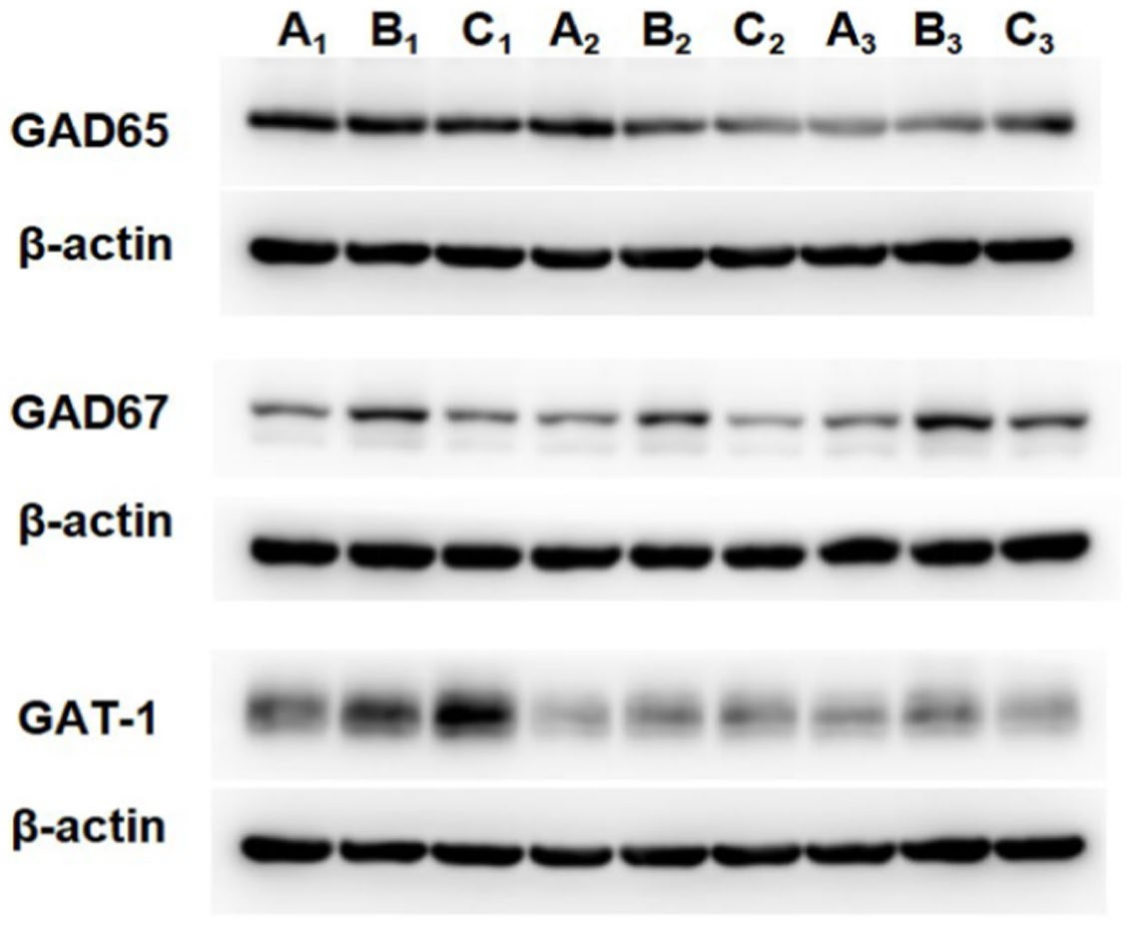
The protein expression of GAD65, GAD67 and GAT-1 in the three groups. A is the Control group, B is the TD group induced by IDPN, C is the Tia group.

The GAD67 protein expression level in the TD group was increased compared to that of the Control group (*p* = 0.009). After treatment, the GAD67 level in the Tia group was lower than that in the TD group (*p* = 0.039). There were no statistically significant differences in the GAD65 and GAT-1 protein expression levels among the three groups. The results are presented in Table 3 and Figures 4, 5.

**Table 3:** Comparison of GAD65, GAD67 and GAT-1 protein expression in the three groups.

Group	GAD65	GAD67	GAT-1
Control group	0.60 ± 0.23	0.23 ± 0.10	0.70 ± 0.25
TD group induced by IDPN	0.58 ± 0.15	0.44 ± 0.15^##^	0.93 ± 0.26
Tia group	0.57 ± 0.12	0.28 ± 0.10*	0.98 ± 0.37

Compared with the Control group, ^#^means *p* < 0.05, ^##^means *p* < 0.01; compared with the TD group induced by IDPN, *means *p* < 0.05, **means *p* < 0.01.

### 3.4. Comparison of mRNA transcription level of GAD65, GAD67, and GAT-1 in “Glu-GABA” metabolic loop

**Figure 6:**
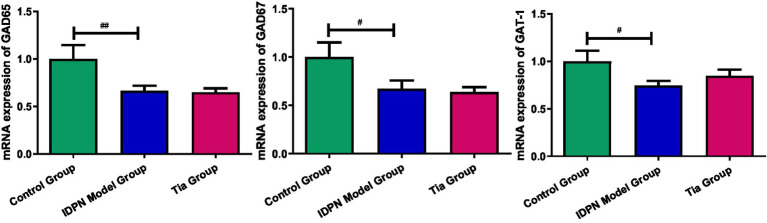
The mRNA expression of GAD65, GAD67 and GAT-1 in the three groups. Compared with the Control group, ^#^ means *p* < 0.05, ^##^ means *p* < 0.01; compared with the TD group induced by IDPN, * means *p* < 0.05, ** means *p* < 0.01.

Compared with that of the Control group, the GAD65 mRNA expression in the TD group was significantly decreased (*p* = 0.022). The GAD67 mRNA expression in the TD group was significantly decreased (*p* = 0.043) compared with that of the Control group; the TD group exhibited significantly decreased GAT-1 mRNA expression compared to the Control group (*p* = 0.041); A significant difference in GAD65, GAD67, and GAT-1 mRNA expression was not observed between the TD and Tia groups. The results are presented in Table 4 and Figure 6.

**Table 4:** The mRNA expression of GAD65, GAD67, and GAT-1 in the three groups.

Group	GAD65	GAD67	GAT-1
Control group	1.00 ± 0.36	1.00 ± 0.37	1.00 ± 0.28
TD group induced by IDPN	0.66 ± 0.13^**##**^	0.67 ± 0.21^#^	0.75 ± 0.12^#^
Tia group	0.65 ± 0.10	0.64 ± 0.13	0.85 ± 0.16

Compared with the Control group, ^#^means *p* < 0.05, ^##^means *p* < 0.01.

### 3.5. Comparison of other metabolites in the thalamus detected using widely targeted metabolomic analysis between TD group and Control group rats

#### 3.5.1. Full mass spectrometric analysis of metabolites

**Figure 7:**
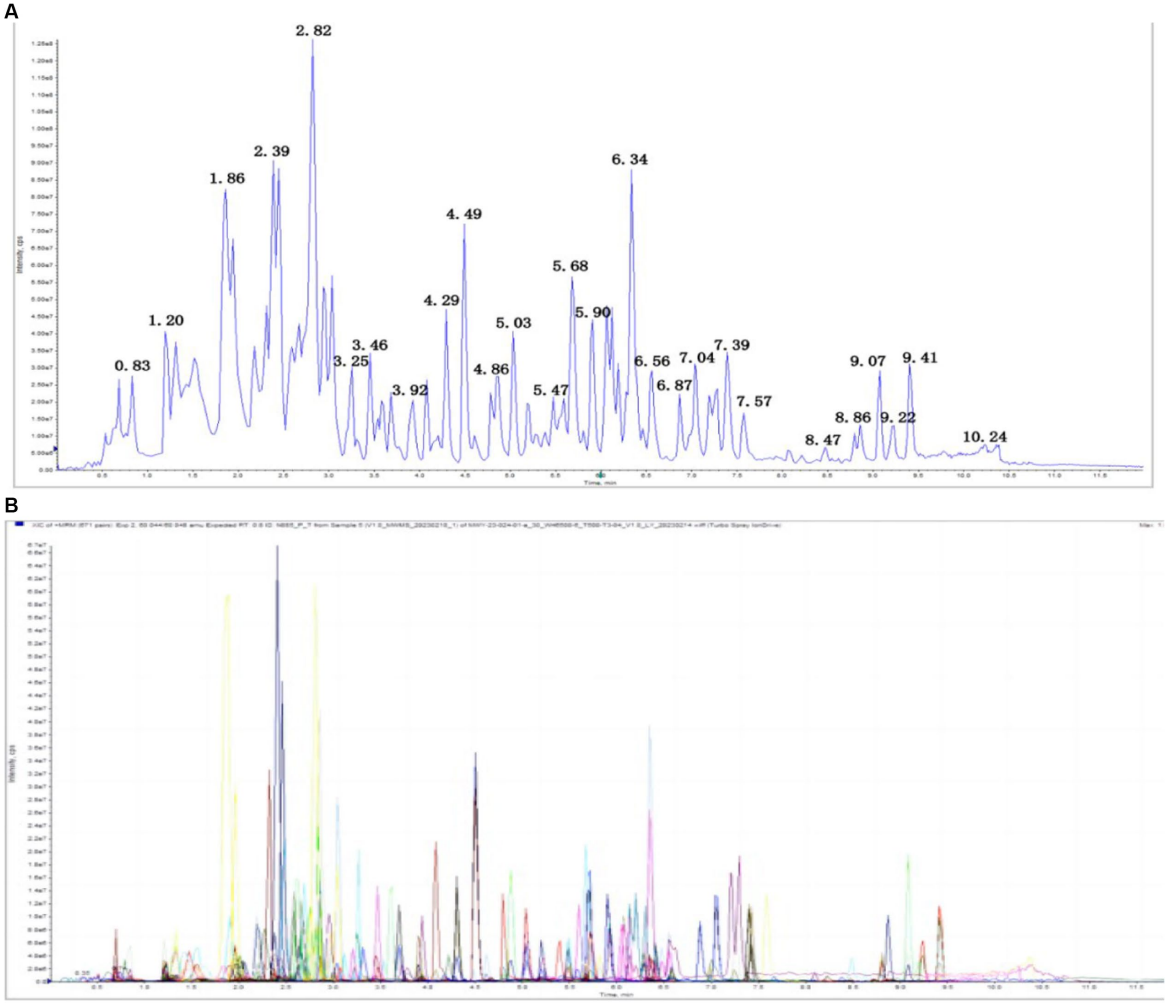
**(A)** Total ion current of one quality control sample, as revealed by mass spectrometry detection. **(B)** A multi-peak detection plot of the metabolites in the multiple reaction monitoring mode.

A total of 242 metabolites were detected, including 47 nucleotides and their metabolites, 24 amino acids, 23 small peptides, 21 organic acids and their derivatives, 21 amino acid derivatives, 12 hormones and hormone-related compounds, 11 CAR, 11 bile acids,10 coenzymes and vitamins, 6 amines, 6 polyamines, five phosphate sugars, four phosphoric acids, three sulfonic acids, three heterocyclic compounds, three phenolic acids, and other metabolites. Overlay analysis of the QC-TIC diagram (Figure 7A) and the sample multi-peak detection diagram (Figure 7B) showed that the instrument performed stably, and the data recorded in this study had good repeatability and reliability.

#### 3.5.2. Differential metabolites selected

Significantly differentially regulated metabolites between groups were determined using variable importance in projection (VIP) and absolute Log2FC (fold change). VIP values were extracted from the partial least squares discriminant analysis (PLS-DA) results, which also contained score plots and permutation 57 plots, and were generated using the R package Metabo AnalystR. The data were log-transformed (log2) and mean-centered before OPLS-DA. A permutation test (200 permutations) was performed to avoid overfitting.

**Figure 8:**
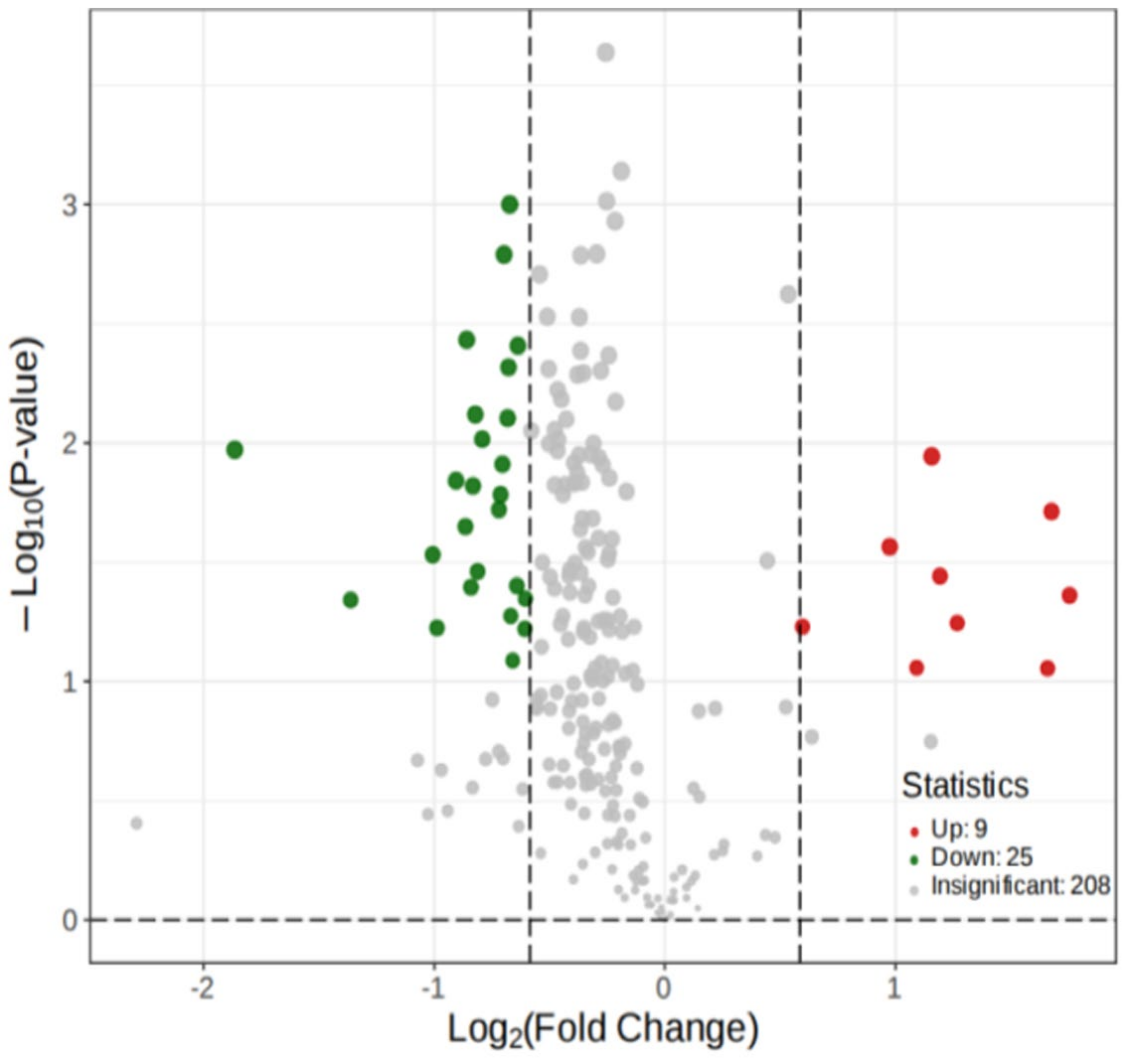
Volcano Plot of the metabolites.

To explore the differences in the metabolites, OPLS-DA was performed, according to the principle of VIP > 1 and fold change ≥2 or ≤ 0.5 for the screening of the different metabolites (Chu et al., 2020). A total of 34 different substances were screened (up-regulated: 9, downregulated: 25). The volcano plots are shown (Figure 8).

**Figure 9:**
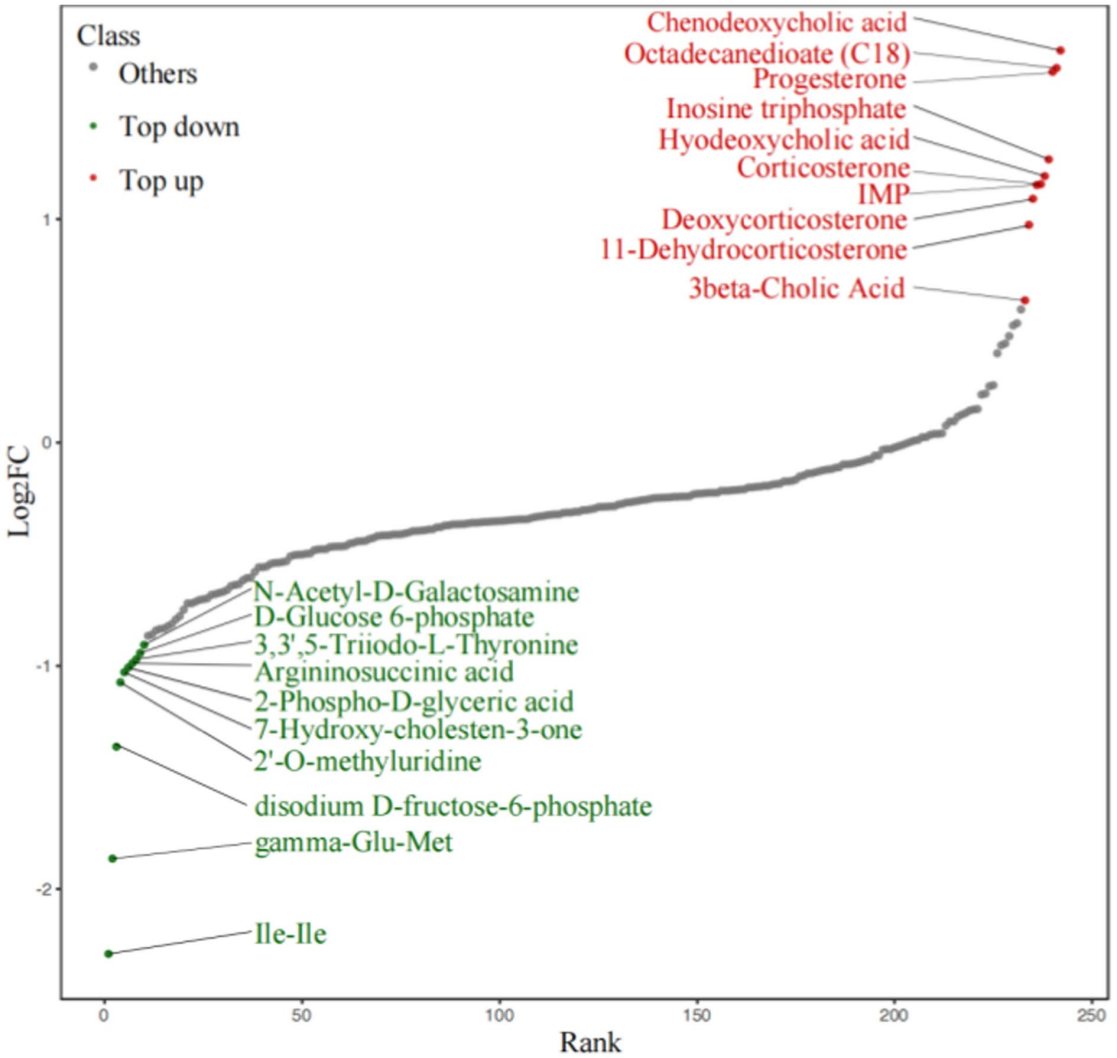
Dynamic distribution of difference metabolite content.

To demonstrate the overall metabolite differences more intuitively, the FC values of the metabolites in the comparison group were calculated. Dynamic distribution maps of metabolite content differences were created based on the arrangement of FC values from low to high (Figure 9), and the top 10 upregulated and downregulated metabolites were labeled. Steroid levels (progesterone, 11-dehydrocorticosterone, deoxycorticosterone, corticosterone) showed distinct differences between the TD and Control group.

**Figure 10:**
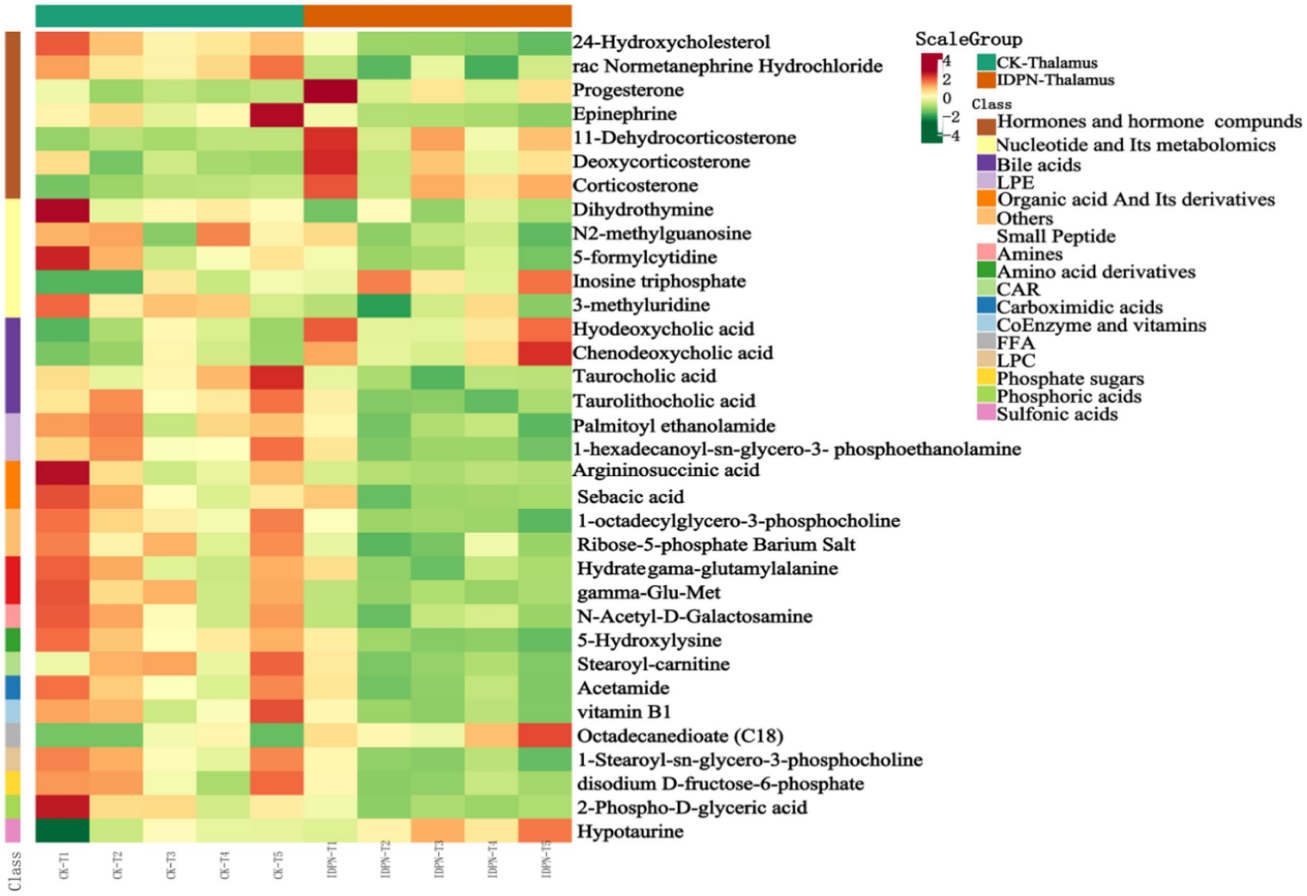
Differential metabolite cluster heat map.

**Figure 11:**
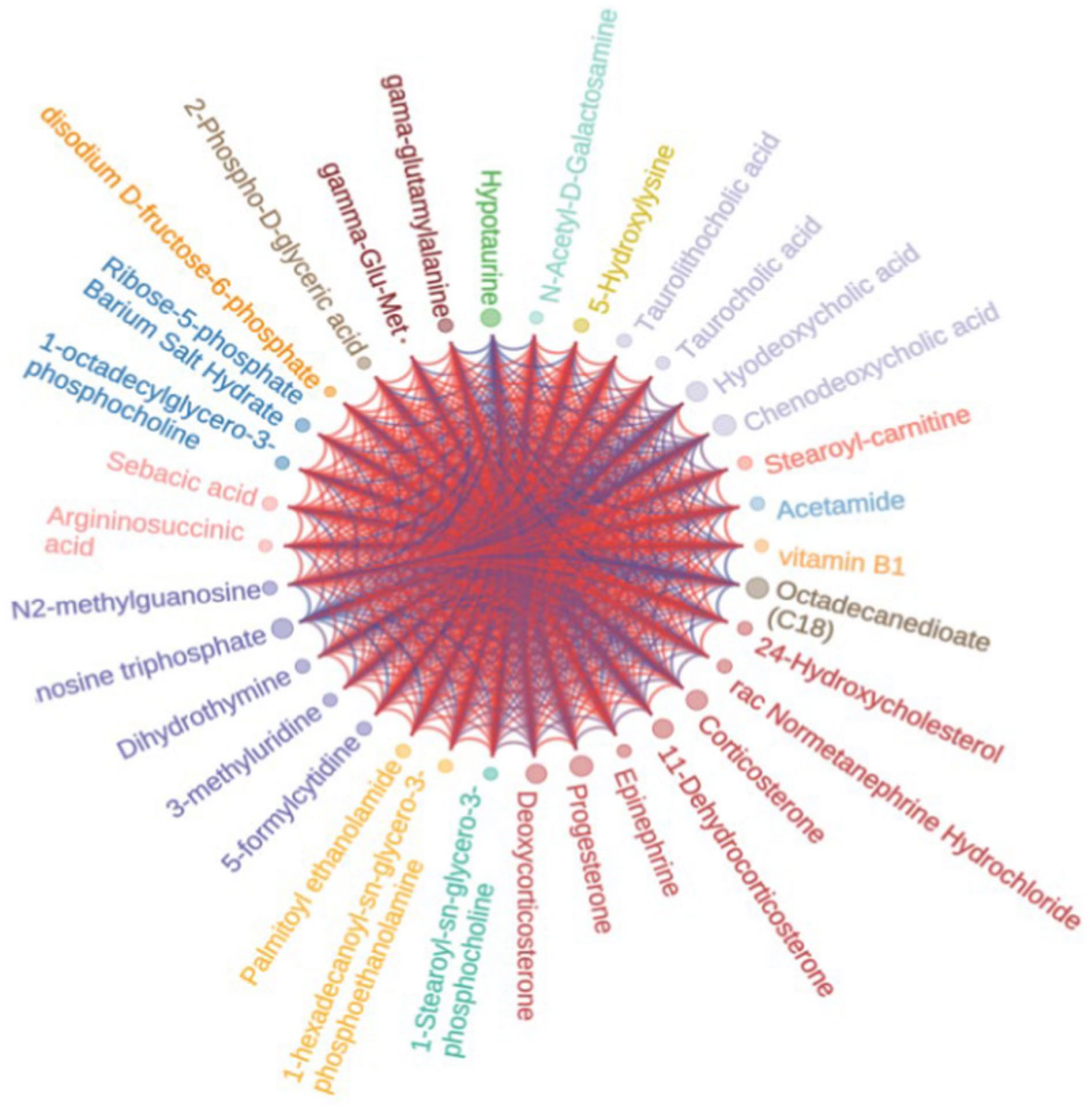
Differential metabolite chord diagram. The size of the dots in the figure represented Log_2_FC value. The color of the dots represented the classification of source of metabolites, and the lines represented the value of phase relationship of metabolites in corresponding positions.

To observe the variation rule of metabolite content, we adopted the United Variance Scaling (UV, Scaling) analysis for the original content of differential metabolites and used R software packages to draw heat maps (Figure 10). We also observed a close correlation between differential metabolites (Figure 11).

#### 3.5.3. Enrichment analysis of differential metabolites Kyoto encyclopedia of genes and genomes

**Figure 12:**
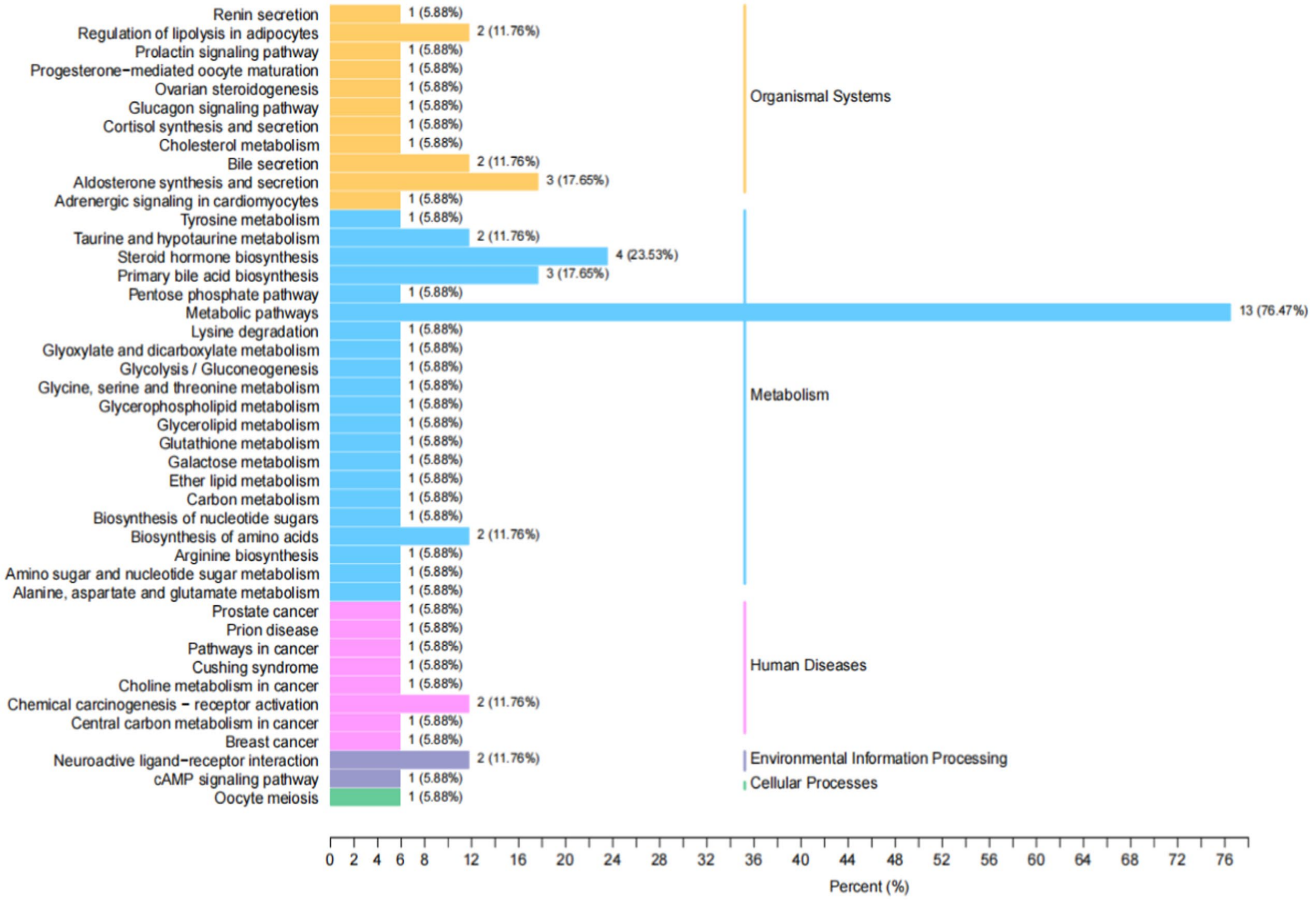
KEGG classification map of differential metabolites. The ordinate is the name of the KEGG metabolic pathway, and the abscess is the number of differential metabolites annotated to the pathway and their proportion to the total metabolites annotated.

Metabolites interact with neurons to activate various pathways. The annotation results of the significantly different metabolites in Kyoto encyclopedia of genes and genomes (KEGG) were classified according to the pathway types in KEGG, as shown in Figure 12. The metabolic pathway was the primary pathway, followed by steroid hormone biosynthesis.

## 4. Discussion

This study is the first to reveal metabolic abnormalities in the thalamus of rats with TD induced by IDPN. Rats with IDPN-induced TD presented with disrupted E/I balance and abnormalities of the “Glu-GABA” metabolic loop. We also found that steroid levels (progesterone, 11-dehydrocorticosterone, deoxycorticosterone, corticosterone) showed distinct differences between the TD and Control group. Therefore, the steroid hormone biosynthesis pathway may be involved in the pathogenesis of TD. Disorders of neurotransmitter metabolism of the thalamus should be considered.

Glutamate is the main excitatory amino acid neurotransmitter that increases neuronal excitement (Reiner and Levitz, 2018). GABA is the main inhibitory amino acid neurotransmitter that antagonizes the effects of Glu and decreases neuronal excitement (Zhu et al., 2018). In rats with TD, increased Glu levels and decreased GABA levels in the thalamus led to an increased E/I ratio. In the “Glu-GABA” metabolic loop, GAD is the rate-limiting enzyme in the synthesis of GABA, and GAT-1 is the main transporter for the uptake of GABA. They all participate in maintaining the amino acid neurotransmitter balance (Cheng and Carvill, 2021; Genčić et al., 2022). In this study, the protein expression level of GAD67 increased, and the mRNA expression levels of GAD65, GAD67, and GAT-1 were decreased. However, these indicators did not help to convert more Glu to GABA to reduce nerve excitability and maintain the balance of neurotransmitters. In addition, the increase in Asp level and decrease in Gly level further increased the excitement of rats. The open field test and stereotyped scores of the rats increased. The behavior of the rats was more pronounced.

The results of PCR showed that the mRNA expression levels of GAD65, GAD67 and GAT-1 in the TD group were lower than those in the Control group; however, western blotting revealed that the protein expression level of GAD67 was increased. They can also occur physiologically. Transcription occurs in the nucleus, whereas translation occurs in the cytoplasm. Spatial and temporal differences exist between transcription and translation. Gene expression can transfer information from DNA to proteins via transcription and translation (Kummer and Ban, 2021). Transcription produces mRNA, whereas translation creates protein. Due to the longer half-life of the protein, it could persist longer and accumulate more than mRNA in cells, causing a difference between the results of western blotting and PCR. In addition, post-transcription occurs between transcription and translation. Gene expression is precisely controlled by both transcriptional and post-transcriptional regulation. Post-transcriptional regulation at the mRNA stability level is essential for gene expression (Lipshitz et al., 2017). Therefore, more studies are required to determine the changes in the post-transcriptional regulation of TD.

Compared to that of the TD group, the Glu level in the Tia group was significantly decreased, and the Glu/GABA ratio was significantly decreased; however, the protein and mRNA expression levels of GAD65, GAD67, and GAT-1 were not significantly different between the TD and Tia group. These results suggest that Tia may regulate the E/I balance via other pathways. Hence, the treatment mechanism of Tia requires further study. Tia did not appear to be involved in the metabolic loop. In the future, it is expected that targeted drugs will be developed to reverse abnormal metabolic circuits, and this study provides an important reference.

We conducted a widely targeted metabolomic study to comprehensively detect and analyze metabolite abnormalities in the thalamus of rats with IDPN-induced TD. We found that a total of 34 different substances were screened (9 were up-regulated, 25 were down-regulated). The upregulated metabolites included four hormones and hormone-related compounds, two bile acids, one free fatty acid, one nucleotide and its metabolomics, and one sulfonic acid. Downregulated metabolites included three hormones and hormone-related compounds, six nucleotides and their metabolomics, one sulfonic acid, and two bile acids. There was a close correlation between the different metabolites. Metabolites interact with neurons to activate various pathways. The annotation results of significantly different metabolites in KEGG were classified according to the pathway types. In our study, the metabolic pathways were first, followed by steroid hormone biosynthesis. Thus, the steroid hormone biosynthesis pathway may be involved in the pathogenesis of TD.

Since the 1980s, it was recognized that certain steroids, termed neurosteroids (Corpéchot et al., 1981), are produced locally in the brain and influence neuronal physiology. Subsequent research revealed the intriguing complexity of neurosteroid signaling: one type of neurosteroid often influences multiple molecular targets, and a single target is often modulated by multiple neurosteroids, sometimes in opposite directions. At excitatory synapses, neurosteroid modulation primarily targets the NMDA-type ionotropic glutamate receptors. NMDA receptor inhibition by neurosteroids can be neuroprotective against excitotoxic neurodegeneration caused by excessive NMDA receptor activation (Joksimovic et al., 2018; Ratner et al., 2019). Neuroactive steroids possess stress-protective properties and modulate the function of GABAA receptors (GABAARs) (Gunn et al., 2015; Joksimovic et al., 2018; Ratner et al., 2019). The neurosteroids that increased in our study may have affected the GABAA or NMDA receptors to influence the balance between excitation and inhibition in neuronal networks. Additionally, progesterone metabolites may enhance Glu release by potentiating GABAARs located in the presynaptic terminal membrane. We intend to confirm this hypothesis in future experiments.

This study had some limitations. The study only involved the thalamus, and pathophysiological changes in other brain regions (such as the cortex) may differ. Therefore, more comprehensive studies are required to better understand the pathogenesis of TD.

## Data availability statement

The raw data supporting the conclusions of this article will be made available by the authors, without undue reservation.

## Ethics statement

The animal study was approved by the Ethics Committee on Animal Experiments of Shanghai Jiao Tong University School of Medicine. The study was conducted in accordance with the local legislation and institutional requirements.

## Author contributions

JH and JY designed the study, completed the animal experiment, and wrote the manuscript. XY and XZ analyzed the data for the manuscript. All authors contributed to the article and approved the submitted version.

## Footnotes

^1^ https://sciex.com.cn/

^2^ https://sciex.com/

^3^ http://www.metware.cn/
